# Designed to deter: Barriers to facilities at secondary schools in Ghana

**DOI:** 10.4102/ajod.v1i1.2

**Published:** 2012-05-16

**Authors:** Anthony K. Danso, Frances E. Owusu-Ansah, Divine Alorwu

**Affiliations:** 1 Department of Building Technology, Kwame Nkrumah University of Science and Technology, Ghana; 2 Department of Behavioural Sciences, Kwame Nkrumah University of Science and Technology, Ghana

## Abstract

**Background:**

There are varied and complex problems associated with the admission of students with disabilities into secondary (senior high) schools all over the world. This situation is further complicated by difficulties encountered in the built environment of these institutions and, in this, Ghana is no exception.

**Objectives:**

This exploratory study investigated the level of accessibility of the built environment in secondary schools in eight out of the ten regions of Ghana, in order to determine whether they conform to guidelines provided in international building standards and also assess the extent to which they have been designed and constructed to meet the provisions of the *Persons with Disability Act 2006*, which allows for equal access to public buildings in Ghana.

**Method:**

In total, 705 building elements in 264 facilities were surveyed using international standards, building codes, regulations and guidelines. These facilities included car parks, classrooms, dormitories, assembly halls, telephone booths and administration blocks.

**Results:**

Our findings revealed that most of the building elements were barring and not disability-friendly. Just to name a few: there were obstructions on access routes to and around buildings, absence of designated car parks, unfriendly vertical and horizontal means of circulation in buildings and lack of accessible sanitary accommodations. In addition, the general lighting and signage were poor. As a result, very few students with disabilities are admitted and retained in these schools.

**Conclusion:**

Mainstreaming of people with disabilities into the Ghanaian educational system remains impossible unless urgent action is taken to alter the facilities at secondary schools. Based on this research outcome, recommendations have been made to the Ghanaian government and the Ghana Education Service, as well as non-governmental organisations and relevant professional bodies for the amelioration of the present situation in our secondary schools.

## Introduction

### Background

Ghana is a middle-income country with a per capita income of $1600 in 2010 and was the first Black African nation to gain independence from British rule in 1957. Located on the west coast of Africa and with tropical climate, it is bounded by three French-speaking countries namely, Cote d’Ivoire, Burkina Faso and Togo to the west, north and east, respectively, and by the Gulf of Guinea to the south. In 1996, the Ghanaian government launched an ambitious pre-tertiary education programme, called the free, compulsory universal basic education (FCUBE) initiative, which sought to make basic (primary and junior secondary) education in Ghana compulsory and tuition-free. Aside from this, expenditure on education by the government has grown steadily from 1.5% of GDP in the early 1980s to about 3.5% in recent years. Despite these laudable initiatives, quality education remains inaccessible to a number of school children, most notably persons with disability.

After many years of military rule, the country returned to constitutional rule and promulgated a new Constitution in 1992. Article 29(4) of the 1992 Constitution of Ghana (Ministry of Justice 2005) avers that persons with disabilities shall be protected against all forms of discrimination that are exploitative, abusive or degrading in nature. However, the Ghana Federation of the Disabled (2008) reports that persons with disability continue to face discrimination in all aspects of their social and professional lives. This has resulted in Ghanaians with disabilities being amongst the country’s most marginalised and poorest inhabitants. To reverse or ameliorate the situation, organisations providing aid or services to people with disabilities joined hands under the auspices of both the Ghana Federation of the Disabled and the Government of Ghana to facilitate the passage of the *Persons with Disability Act* 2006 (PDA) (*Act No. 715 of 2006*) (Republic of Ghana 2006). The PDA was aimed at enabling persons with disability to enjoy rights enshrined in the Constitution, with the view to improving their living standards and mainstreaming their activities. These rights included, amongst others, accessibility to all public places, education, health care, transportation, recreation, equal employment opportunities and the creation of special bureaus at employment centres specifically for persons with disability. This study covers the education of persons with disability in secondary schools in eight out of the ten regions of Ghana. Findings are expected to educate, challenge and sensitise the general public to the needs of persons with disability in educational facilities and assist in the removal of those barriers that exclude them from the mainstream educational system of Ghana.

### Literature review

#### Discrimination against people with disabilities

There is ample evidence to suggest that people with disabilities face discrimination in most spheres of their daily lives (Gleeson 2001; Imrie & Hall 2001). The discrimination that the 1992 Constitution (Ministry of Justice 2005) and the PDA of 2006 (Republic of Ghana 2006) sought to eradicate comes in various forms. The social perception that disability equals inability and, thus, people with disabilities are incapable of making a meaningful contribution to national development is one of the many forms of discrimination (GFD 2008). Another is the stigmatisation and perception that they are dependent and not part of the ‘normal’ society because of their disability (Oliver 1990).

The discrimination against people with disabilities extends to the built environment, where it is reported that about 90% of all individuals may become architecturally inhibited in some way at some point in life because of the inappropriateness of the design and construction of building elements such as narrow doorways, stairs and complex door furniture (Wylde, Baron-Robbins & Clarks 1994). Designers of the built environment often seek to make it more accessible to all through the concept of universal design, which involves ‘designing buildings that are suitable for all users, and the removal of inadequate or inappropriate design solutions to disable users’ (Imrie & Hall 2001:335). Although much of the work in this area was concerned initially and primarily with the needs of people with disabilities, there is now a move towards discussing universal design in terms of all end users. Many people recognise the need to include the end user in the building design process and so shift the designer’s focus from one of pure aesthetics to functionality. It is also a fact that people with disabilities, through their daily experiences, are able to develop better insights of their architectural needs than most architects (Heylighen, Michiels & Van Huffel 2006). As Imrie and Hall (2001) argue, this:

experiential knowledge of people with disabilities is critical in the shaping of building design and assisting with the formulation and direction of subsequent changes that keep the environment dynamic and responsive to changing needs. (Imrie & Hall 2001:337)

#### Regulatory frameworks and international building instruments

As observed earlier, one of the challenges facing people with disabilities is ease of access to the built environment. A number of countries, particularly in the Western world, have in recent years enacted regulatory framework in the forms of persons with disability legislations: planning and building regulations which require public buildings to be accessible (Varol & Erco kun 2006). In order to monitor and enforce accessibility in public buildings, building instruments are required to assess the standard of accessibility that should be achieved. A number of these instruments have been developed over the years (Mace 1998) and they include the British Standard 8300 (BS8300) (BSI Group 2001), Americans with Disabilities Act Accessibility Guidelines (ADAAG) (United States Access Board 1990) and a joint product by the United Kingdom, United States of America and Lebanon (Solidere 2004). The main goal of these standards is to provide guidance on how the built environment can be designed to anticipate and overcome restrictions that prevent people with disabilities from making full use of the premises and their surroundings. Typically, they provide a checklist of items that specify measurements which need to be met in order for the item to be accessible. Examples of such items are the height of steps (risers), the depth of steps (goings), slope of ramps (gradient) and the type and quantity of sanitary appliances, et cetera. Their recommendations include, but are not limited to, elements of construction and accommodation in general, specific building types and the management and maintenance for safe access and use by people with disabilities. Although it took long and tortuous research to craft them, some of these international building instruments do still contain some deficiencies (Feeney 2003). For example the BS8300 over-stresses restricted mobility, with little attention given to the needs of people with sensory and cognitive impairments (Bichard, Hanson & Greed 2006).

The Ghana Education Service (GES) has an all-inclusive policy that seeks to provide education for all school children, including students with disabilities, and this has led to the establishment of specialist secondary schools such as Okuapeman Senior High School, Wa Senior High School for the Deaf and Mampong Akwapem Senior High Technical School. The aim was to provide a congenial, safe and an all-inclusive environment and specialist training for the students in these schools. However, the reality is that the built environments in most of these specialist schools include buildings which are not disability-friendly, making the establishment of these schools redundant. Furthermore, these specialist schools are not given the requisite financial, material and human resources needed for their specialist training. This often results in their abysmal academic performance, which is evidenced by the fact that none of the specialist secondary schools in the country belongs to the list of elite secondary (Category ‘A’ and ‘B’) schools provided by the GES. The quest for higher education by students with disabilities is therefore made more daunting because, apart from their physical disabilities, they are expected to compete on merit with students from the traditional elite secondary schools that have better facilities for placement into tertiary institutions in the country. It is therefore not surprising that very few of these students gain admission into tertiary institutions in Ghana. For this reason, in their quest to get quality education for their children with disabilities, most parents in Ghana prefer enrolling these children in traditional secondary schools.

One way of addressing this imbalance is to provide an accessible environment in some, if not all the traditional secondary schools. The present condition of these schools has also heightened the perception of neglect, discrimination and stigmatisation by successive governments. This perception has been deepened further by the fact that no attempts have been made since the passage of the PDA in 2006 to revise the *Draft Ghana building code* (Council for Scientific and Industrial Research 1988) and *National building regulations* (Ministry of Works and Housing 1996) which regulate the construction of buildings in Ghana to include the concept of universal designs. As a nation, Ghana therefore does not have a policy framework that regulates and obliges the stakeholders in the building industry to design and build structures that are disability-friendly. These anomalies motivated this research so as to contribute to the development of social consciousness with respect to the equal participation of persons with disabilities in secondary education in Ghana.

#### Aim and objectives of study

The aim of this study therefore was to determine whether buildings and facilities on the campuses of Category ‘A’ senior high schools in Ghana are barrier free and disability-friendly. Specific objectives include comprehensive comparison of design in the built environment in secondary institutions in Ghana to international standards and building instruments. It is our belief that through our findings we can bring to the fore the need for revision, enforcement and regulatory mechanisms in building designs to facilitate mainstreaming in secondary education in Ghana.

## Research method and design

### Materials

Three international standards and building instruments, namely the BS8300 (BSI Group 2001), the ADAAG (USAB 1990) and Solidere (2004), were used to compile checklists and questionnaires for the quantitative measurements and the subsequent interviews. Quantitative measurements, taken from the observed buildings, were compared to these standardised building instruments. Qualitative data focused on the accessibility of the built environment, as stipulated in the abovementioned instruments, were obtained from interviews with the relevant stakeholders and institutions.

### Setting and design

For the task of data collection and subsequent data analyses, a descriptive, cross-sectional methodological approach was employed for this survey research. Data were collected between 2008 and 2010 by final-year students of the Department of Building Technology at the Kwame Nkrumah University of Science and Technology, Kumasi, under the directive of the corresponding author. To ensure consistency, all research assistants were trained on the procedure of data collection, which included integration of both qualitative and quantitative methods.

### Sample and data collection method

The population for the study was the number of Category ‘A’ public senior high schools in Ghana, which stood at 65 in 2010. The GES has categorised these schools into four groups, with Category ‘A’ schools being the most endowed in terms of academic performance and physical infrastructure (Table 1). Twenty-one of the Category ‘A’ secondary schools were selected from eight out of the ten regions of Ghana using non-random purposive probability sampling which was triggered by, amongst other things, the classification of the GES, the year of establishment, academic performance, student population, location, popularity and, in some cases, the number of prominent products produced in the country (Table 2).

**TABLE 1:** Categories of public senior high schools (*N* = 502) in Ghana.

Category	*n*	%
A	65	13.0
B	72	14.3
C	170	33.9
D	195	38.8

*Source*: Ghana Education Service, 2011, ‘Computerized school selection and placement system (CSSPS) guidelines for selection of schools for placement’, *Ghana Education Service Register of Programmes for Public Senior High School*, GES, Accra. *n*, Given as number of schools.

**TABLE 2:** List of schools surveyed in this study.

Name of school	Region	Year founded
Presbyterian Boys Secondary, Accra	Greater Accra	1938
Achimota Secondary School, Accra	Greater Accra	1927
Accra Girls School, Accra	Greater Accra	1960
Mfantsipim School, Cape Coast	Central	1876
Adisadel College, Cape Coast	Central	1910
Opoku Ware School, Kumasi	Ashanti	1952
Prempeh College, Kumasi	Ashanti	1947
Yaa Asantewaa Girls Secondary School, Kumasi	Ashanti	1960
Ghana Secondary Technical School, Takoradi	Western	1909
Archbishop Porter Girls Secondary School, Takoradi	Western	1965
Bishop Herman College, Kpando	Volta	1952
Mawuli Senior High School, Ho	Volta	1950
Our Lady of Apostles Senior High School, Ho	Volta	1954
Our Lady of Apostles Girls Senior High School, Kenyasi	Brong Ahafo	1974
Sunyani Senior High School, Sunyani	Brong Ahafo	1960
St. James Seminary & Senior High School, Sunyani	Brong Ahafo	1978
Tamale Senior High School, Tamale	Northern	1951
Ghana Senior High School, Tamale	Northern	1960
Bolgatanga Girls Senior High School, Bolgatanga	Upper East	1956
Navrongo Senior High School, Navrongo	Upper East	1960
Notre Dame Minor Seminary/Secondary School, Navrongo	Upper East	1960

A survey, which involved a one-time observation of randomly selected buildings and facilities in each school, was conducted on their level of compliance as per the building standards used. The breakdown of the gender of the selected schools is shown in Table 3. A total of 705 elements in 264 buildings and facilities were surveyed in the schools and measurements were taken to the nearest 5 mm (Table 4). Questionnaires that centred on the number of students with disabilities and employees in each school, the approximate age of the buildings and the school’s policy on the admission of students were administered to the authorities of all the schools, out of which 14 were completed. In addition to the questionnaires, authorities in 11 schools were interviewed on some aspects of the questionnaires to buffer findings. Authorities of the remaining 10 schools were either not available at the time of the interviews or refused to be interviewed.

**TABLE 3:** Distribution of schools by gender.

Gender	Category ‘A’ schools in Ghana		Category ‘A’ schools selected		Category ‘A’ schools used	
	*n*	%	*n*	%	*n*	%
Boys only	14	21.5	9	42.8	-	64.3
Girls only	15	23.1	6	28.6	-	40.0
Mixed	36	55.4	6	28.6	-	16.7
**Total**	**65**	**100**	**21**	**100**	**-**	**-**

*n*, Given as number of schools.

**TABLE 4:** Type and number of buildings or facilities surveyed.

Type of building or facility	*n*	%
School car parks	22	8.33
Classroom blocks	36	13.64
Dormitory blocks	72	27.27
Administration blocks	24	9.09
Church buildings or mosques	16	6.06
Dining halls	24	9.09
Assembly halls	21	7.95
Laboratory blocks	10	3.79
Libraries	12	4.55
Playing fields	5	1.89
Public telephones	22	8.33
**Total**	**264**	**100**

*n*, Given as number of surveyed facilities.

### Analysis

The data gathered were analysed to determine the level of compliance of each element to the various buildings and facilities in the schools. The checklist and the questionnaire used for the auditing of the facilities and buildings were abridged from the requirements of the international instruments. The requirements were grouped under nine main elements (Table 4) and the barriers within each element were graded from 1 to 4 (1 = no restriction, 2 = mild restriction, 3 = moderate restriction, 4 = complete restriction) by the corresponding author, based on that element’s level of compliance or restrictions in comparison with the standards. For ease of analysis, the barriers identified were further categorised into two main groups: none to mild restrictions and moderate to severe restrictions.

## Results

A total of 705 elements from 264 buildings and facilities in 21 Category ‘A’ senior high schools in Ghana were surveyed. Each element was examined as per the requirements of the international instruments which were organised into nine main groups (see Online Appendix). The results indicated that in most institutions there were moderate to severe restrictions in seven out of the nine elements; except in signage and audible communication, where the level of compliance ranged from no restrictions to mild restrictions (Table 5).

**TABLE 5:** Level of compliance of elements in relation to international instruments.

Elements	No restriction		Mild restriction		Moderate restriction		Severe restriction		Total	
	*n*	%	*n*	%	*n*	%	*n*	%	*N*	%
Car parks	-	-	-	-	2	9.1	20	90.9	22	3.1
Access routes	-	-	2	8.0	8	32.0	15	60.0	25	3.6
Vertical circulation: staircases, ramps and lifts	3	3.0	18	18.2	41	41.1	37	37.4	109	15.5
Horizontal circulation and entrances	14	9.7	32	22.2	53	36.8	45	31.3	144	20.4
Signage and information	83	49.4	64	38.0	6	3.6	15	8.9	168	23.8
Audible communication	16	69.6	5	21.7	2	8.7	-	-	23	3.3
General lighting	27	16.1	35	20.8	49	29.2	57	33.9	168	23.8
Public telephone	-	-	-	-	7	31.8	15	68.2	22	3.1
Sanitary accommodation	-	-	-	-	4	16.7	20	83.3	24	3.4
**Total**	**-**	**-**	**-**	**-**	**-**	**-**	**-**	**-**	**705**	**100**

*n*, Give as number of elements; *N*, Given as total number of elements.

### Car parks and garages

All the schools surveyed had car parks which were used by both staff and visitors. The results (Table 5) indicated that only 9.1% of the car parks had moderate restrictions and 90.9% had severe restrictions. This was due to the fact that apart from the absence of designated car parks for persons with disability in all the schools, a greater number of the existing car parks had uneven bituminous surfaces with potholes that were neither marked nor signposted. Besides this, car parks of some senior high schools in the Upper East Region had lateritic (unpaved) surfaces which turned muddy during the rainy season.

### Access routes to and around buildings

On the contrary, the schools in the Ashanti Region generally had well laid out concrete or bituminous walkways that linked most of the buildings on their campuses. A school in the Western region also had an oval-shaped tarred road network that linked all eight dormitories and other facilities in the school.

#### Vertical circulation: Staircases, ramps and lifts

Vertical circulation refers to the vertical movement of people from one floor to another within or between buildings and facilities. Building components that are usually employed to scale these heights include staircases, ramps and lifts. Most of the buildings surveyed in this study were either two-storey (71%) or three-storey (3%) that were mostly accessed by staircases. A few of the staircases were complemented by ramps but none of the ramps had hand rails. For instance, only one ramp was seen in all the three schools surveyed in the Greater Accra Region. In all, only 10 ramps had been provided in all the 264 facilities, of which 3 were accessible and 7 had moderate to severe restrictions. A major restriction of the ramps was that the slopes were steeper than 1:12, the gradient ratio required by the building instruments. Also, whilst most staircases (94%) had adequate widths, uniform risers (height of a step) and goings (depth of a step), a few (9%) had no handrails.

None of the schools had lifts or elevators, although it was required that all public buildings higher than one storey should have them. Secondly, to facilitate the movement of wheelchairs, thresholds (differences between outdoor and indoor levels) should be bevelled and not be higher than 20 mm. Nine out of ten entrances and doorways could not meet this specification. On the whole, 79% of the elements under vertical circulation had moderate to severe restrictions, whilst 21% had none to mild restrictions (Table 5).

#### Horizontal circulation: Entrances, corridors, verandas and floor surfaces

The results of the building elements used for horizontal circulation (building entrances, verandas, corridors and floor surfaces, etc.) in the schools revealed that 68% of these elements were classified as having moderate to severe restrictions and 32% had none to mild restrictions. Most building entrances and doors had colours that contrasted with their backgrounds and this enhanced identification. Widths of doors were also generally adequate; at least 900 mm for single doors and over 1500 mm for double doors. Most doors also had mortise locks with lever handles. Compared with door handles with round knobs, the lever handles were easy to grip and therefore suitable for people with weak grips. For instance, 75%, 50% and 83% of the doors in the three Volta Region schools, respectively, met this ADAAG (USAB 1990) specification. On the contrary, most of the building entrances had thresholds that were higher than 20 mm and were bridged with steps without ramps. Floor finishes of most buildings were made of sand and cement screed although a few had polished terrazzo floors. In addition, most buildings had wide and straight corridors with level and slip-resistant surfaces and no obstructions.

#### Signage and information

Signage is very important in the built environment for easy identification and problems identified with signage by the building instruments included orientation difficulties resulting from illegible directional signs, building or room names and numbering and/or lack of them. Others are pedestrian accidents and hazards that can result from the absence of or badly positioned signs and non-identification of access routes and accessible facilities in the built environment. About 87% of signage provided in the schools met the requirements of BS8300; that is, they had none to mild restrictions. In these cases, the signs and inscriptions were visible, clear, simple, and easy to read and understand. Contrasting colours were also employed to differentiate letters from their backgrounds. On the contrary, some signs in some of the schools surveyed were faded or were written in chalk which got washed away by the rains. Dormitories were the most signed buildings in the schools. A school in the Western Region had the names of all its dormitories signed boldly on their entrances. On the other hand, classrooms, laboratories, dining halls, libraries were rarely signed. The layout of one of the Kumasi schools was mounted near the main school entrance to direct first time visitors to their final destinations on the campus.

#### Audible communication systems

The main forms of audible communication in the schools were centralised sirens and mechanical bells which were sounded at scheduled times of the day to call or signal students at the various parts of their campuses. These communication systems were complemented with public address systems when students congregated at assembly halls, churches and dining halls. Almost 91% of these devices had none to mild restrictions and only 9% had moderate restrictions (Table 5). Audible communication systems had the highest compliance amongst the nine items studied in the survey because most of them were functional; that is, they could be heard loudly and clearly at all the important locations in the various schools.

#### General lighting

General lighting constituted 24% of all items surveyed in the 264 facilities and it refers to natural and/or artificial lighting in the built environment. About a third (34%) of the facilities studied had severe restrictions, whilst 24% were without restrictions (Table 5). It was also observed that natural lighting was generally adequate in all buildings and facilities of the schools because most buildings were carefully oriented to take advantage of sunlight during the day. In the few cases where natural lighting was inadequate, it was complemented by artificial lighting. However, the situation was different at night. The external environment of the schools such as car parks, walkways and access routes were either dark or dimly lit. Only a third of all facilities surveyed had well lit internal spaces at night. Some corridors, classrooms and dormitories were dimly lit, a situation that compelled even good-sighted students to strain their eyes to study or move about. Partially or completely deaf students could not engage in lip reading under such circumstances.

#### Public telephones

**FIGURE 1:**
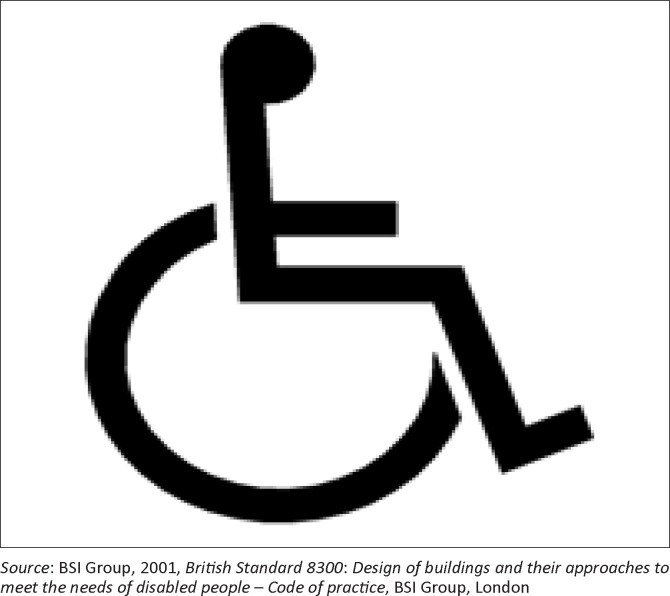
The international symbol of access

For public telephones to remain accessible, the building instruments require that they are provided at accessible locations at suitable heights. Additionally, the telephones should be angled so that they can be used by people when seated and for the visually impaired persons, telephones should have well lit keypads, large embossed or raised numerals that contrast in colour and luminance with their background. Furthermore, the directions to and from these telephones should be clearly marked by combining the international symbol of access (Figure 1) and a telephone symbol (BSI Group 2001).

The survey indicated that 14% of the schools did not have any public telephones and there was only one public telephone in a school in the Upper East Region at the time of the survey. For schools that had them, 100% of the public telephones had moderate to severe restrictions. These restrictions resulted from the fact that none of the phones had directional signs, induction couplers and well lit pads with raised numerals. Moreover, all the receivers were mounted at heights that were beyond the reach of wheelchair users.

#### Sanitary accommodation

The results of the survey (Table 5) revealed that people with disabilities faced severe restrictions in 83% of all restrooms in the senior high schools in Ghana. Not a single sanitary accommodation out of the total of 24 surveyed could be classified as having none or mild restrictions. The 17% of restrooms that were considered as having moderate restrictions for persons with disability were mainly bathrooms which had reasonably wide doors, non-slip floors, wide cubicles and taps fitted at the required heights. The following were examples of impediments observed at the existing sanitary accommodations in the schools:

- There were no restrooms designated for persons with disability in all the schools and the existing restrooms had no vertical and horizontal grab rails.
- Doorways of the existing restrooms were not wide enough to admit wheelchair users and there were insufficient spaces in the cubicles for manoeuvring by wheelchairs users.
- Entrances to the restrooms were, in some cases, rigged with steps and other obstacles.
- A school in the Brong-Ahafo Region only had pit (the Kumasi Ventilated Improved Pit) latrines, which were not suitable for persons with disability.
- Some schools in the Western Region had cubicles with high level cisterns.

## Summary of findings from questionnaires and interviews

Below are the highlights of responses gleaned from interviews with some school authorities:

- Almost all the buildings (98.9%) surveyed were built before 2006; that is, before the PDA (Republic of Ghana 2006) came into being in Ghana.
- No attempts have been made so far to alter and make these buildings and facilities accessible to persons with disability.
- As a policy, the majority of these schools did not admit students with disabilities.
- Only 15 students with disabilities were found in the 21 schools and most of them were made to stay in dormitories and attended classes in classrooms that were located on the ground floors of buildings.
- Only five employees with disabilities were counted in the 21 schools.
- Some physically fit students had parents with disabilities who visited their wards at the schools. Other people with disabilities who were not students, employees of the schools or parents also visited the schools from time to time.

## Ethical considerations

This study was approved by the Department of Building Technology at the Kwame Nkrumah University of Science and Technology. All authorities in the 21 schools selected for this research gave consent for the data gathered in their schools to be used for the research work and its subsequent publication.

### Recruitment procedures and data protection

The mode of data collection and analysis was in accordance with national and international standards. For instance, no student or school authority was obliged to give information. Only those who gave their consent constituted the subjects for the study. Interviews were conducted on a one-to-one basis and numbers instead of names were used for identification during the compilation and analysis of data to ensure the anonymity of the respondents.

### Potential benefits and hazards

Because of the abovementioned data protection procedures, no risks to the subjects are anticipated. Rather, it is the belief of the authors that findings from this research work will galvanise the social and political leaders into providing a more accessible built environment in secondary schools in Ghana.

## Trustworthiness

### Reliability

Apart from the measures mentioned under the ‘Sample and data collection method’ section, only groups of final year students of the Department of Building Technology who had adequate knowledge of the built environment and had been trained in data gathering were used in the survey. The groups, which were made up of 2–4 students, visited each school to take physical measurements, make observations of the elements studied and interview the subjects. They were sent in groups so that they could serve as a check on each other. Secondly only heads of schools and final year students of the secondary schools were interviewed. The abovementioned measures ensured the reliability of the study.

### Validity

The findings and recommendations of the study were validated by some professionals of the built environment (architects, engineers and planners) in Ghana. Also, as noted in the ‘Discussion’ section below, some of the results agreed with findings from similar studies in other countries.

## Discussion

The absence of designated parking spaces in the car parks of the schools, coupled with the poor nature of the existing car parks, completely excluded persons with disabilities from using the of car parks. The state of the existing car parks was in sharp contrast to the requirements of the BS8300 (BSI Group 2001), ADAAG (USAB 1990) and Solidere (2004) standards, which specified that designated public parking spaces should be provided for both employees and visitors with disabilities to a workplace and that they should be differentiated from spaces designated for other users. Furthermore, all uncovered designated parking spaces should be located on firm and level ground with uniform and smooth surfaces and should be as close as possible to the main and all other accessible entrances of the building that uses the parking space. Finally, all car parks with capacity for less than 50 cars should have at least one accessible parking space whose width should be more than 3.60 m.

Access routes in the majority of the schools, especially those in the Northern and Upper East Regions, were unpaved and highly inaccessible. These results compare favourably with the results of a similar study on public buildings that included educational buildings in Ibadan, Nigeria , where Hamzat and Dada (2005) reported that 18.4% of buildings, 45.1% of the building entrances and 19.4% of the access routes were wheelchair accessible. The slight difference between the results of that study and the present one may be attributed to the type of disabilities covered in the two studies; this study covered orthopaedic, visual, hearing, tongue-speech and mental impairments, whilst Hamzat and Dada (2005) investigated only orthopaedic impairments particular to wheelchair users.

The only exceptions to the poor access routes were the schools in the Ashanti Region, where the access routes enhanced the easy movement of wheelchair users and other ambulant (persons who walked with the help of walking aids) persons in these schools. The BS8300 (BSI Group 2001) requires that access routes to and around buildings should be spacious and free from barriers, restrictions and other hazards that could impede free movement, because the provision of narrow approaches creates difficulty for persons with disability. Modification of some of the inaccessible access routes in some schools is therefore required to provide the requisite firm, durable and safe surfaces.

The fact that the majority of the schools have policies that excluded students with disability from gaining admission to the schools do not absolve them from blame if their built environment, especially their car parks and access routes, are not disability-friendly. This is because the survey showed that some schools employed people with disabilities. Furthermore, some visitors and parents of students who visited the schools from time to time also had disabilities. This makes it even more imperative for all the schools to make their facilities more accessible to the entire public.

The building instruments intimated that design problems normally associated with vertical circulation in buildings include differences between indoor and outdoor levels, steep and poorly designed staircases that hinder foot movement, unsafe railings, hard to grip handrails, poorly designed ramps, as well as lifts with inadequate cab space, narrow entry doors, high position of control buttons and short opening intervals. In the absence of lifts, the instruments specified that ramps should be provided whenever stairs obstruct the free passage of pedestrians. As indicated in the results, most schools defaulted on many of the abovementioned specifications. The problem of vertical circulation was felt more keenly in schools attended by students with disabilities, where arrangements had been made for these students, especially wheelchair users and other ambulant persons, to be located on ground floor dormitories and classrooms. This arrangement did not ameliorate the problem completely because other important school facilities such as laboratories, libraries and offices were located above the ground floors of buildings, which made them inaccessible to students with disabilities.

On signage and information, the results indicated that, generally, most schools did not meet the requirements of the building instruments which specify that accessible spaces and facilities should be identified by the international symbol of accessibility, which consists of a wheelchair figure with either a square background or a square border (Figure 1). Contrasting colours are also required to differentiate the figure from the background. It is of interest to note that this symbol was not found on any of the 705 elements in all the 21 schools surveyed and this buttresses the point that most buildings and facilities in the schools were inaccessible to persons with disabilities.

The role of good natural and artificial lighting in ensuring that visually impaired people are able to use buildings conveniently and safely cannot be overemphasised. The luminance on interior surfaces, the quality of the lighting, good colour rendering and the avoidance of glare are key requirements. Older persons and people with visual problems are more sensitive to glare than younger persons. Where one-to-one communication is important, for example between a teacher and a student, it is recommended by the BS8300 (BSI Group 2001) that lighting should illuminate the face of the person speaking to make it easier for lip reading. The results revealed that the schools generally had poor artificial lighting. Remedial measures to improve artificial lighting, especially for the poorly sighted students in the schools, could include replacement of burnt electrical bulbs, installation of additional lights and fixing of street lights.

Telephones in various forms have become one of the most used systems of communication worldwide. The situation is similar in Ghana, where 17 436 949 (72%) and 277 897 (1%) people out of a total estimated population of 24 339 840 people owned cell phones and fixed lines, respectively, in 2010 (CIA World Factbook 2011; National Communications Authority 2011). The 21 schools surveyed had a total of less than 50 fixed line telephones between them for a total student population of over 40 000 students, which makes the density of fixed lines in the secondary schools very worrying when compared with the national density. The situation is even direr when one considers the fact that the use of cell phones is banned in all secondary schools in the country by GES regulations, which leaves the fixed lines as the only means of communication for the students. As a matter of urgency, the telecommunication companies and school authorities should therefore work together to ensure that more public telephones are not only installed but made accessible in the various schools to rectify this situation.

According to the building instruments, problems associated with restrooms for persons with disabilities include insufficient space inside the restrooms, poor design and positioning of fittings and fixtures and sanitary appliance controls that are difficult to grab. In their studies, researchers in the UK and Malaysia (Bichard *et al*. 2005; Rahim & Samad 2010) discovered that public toilets were not accessible to persons with disability in their countries and this severely restricts usage by them (Kitchen & Law 2001). From the results of the present study, the exclusion of persons with disability from sanitary accommodation is also prevalent, even in the best secondary schools in Ghana. The situation should be amended because students cannot stay in the schools without using the restrooms. School authorities will therefore seriously have to consider modifying, altering and reconfiguring their restrooms to make them universally accessible to all students.

As noted earlier, most of the buildings surveyed were built prior to the passing of the PDA in 2006 (Republic of Ghana 2006). Thus their designers probably did not deem it necessary to make the built environment 100% accessible to people with disabilities. Notwithstanding, no attempts have since been made to alter and make these buildings and facilities accessible to people with disabilities, a fact which the school authorities attributed to lack of funds from the central government. As a matter of urgency, the central government should make available funds for this purpose to give meaning to its policy of providing universal education to its citizens. Also, as noted earlier, Ghana as a nation does not have a policy framework that regulates and obliges the stakeholders in the building industry to design and build structures that are disability-friendly. With the passage of the PDA (Republic of Ghana 2006), the *Draft Ghana building code* (CSIR 1988) and the *National building regulations* (Ministry of Works and Housing 1996) should be revised and backed by laws to make it mandatory for all public buildings to be accessible to people with disabilities. It must be pointed out that nothing is gained by legislation without enforcement. The government must therefore have the political will to enforce this law through the relevant state agencies such as District, Municipal and Metropolitan Assemblies and, where necessary, punish all defaulters.

## Recommendations

Based on the above discussion, the following recommendations are made to ensure the accessibility of the above school buildings by persons with disability:

- *Retrofitting*: Facilities in all senior high schools should be improved or altered to meet the internationally accepted standards of a barrier-free educational environment. Elements such as car parks, access routes, door widths, staircases, and public telephones should be altered and others, such as ramps, underfoot warnings, Braille texts, grab rails in restrooms, signage and street lights, should be provided to facilitate the use of the school environment by people with disabilities, including students, employees and visitors. The government, as the owner of most of these public buildings, should provide budgetary allocations for this exercise.
- *Workshop and seminars:* Major stakeholders such as the government, policymakers, non-governmental organisations and persons with disability groups under the Ghana Federation of the Disabled should collaborate and organise workshops and seminars to educate the general public on the need for barrier-free built environments. Public education through the electronic and the print media can also be carried out. These activities will sensitise the general public, who are mostly oblivious to the needs of persons with disabilities in society, and this will eventually accelerate the rate of integration of these persons into mainstream society.
- *Professional institutions*: Professional institutions of the built environment, such as the Ghana Institute of Architects, the Ghana Institution of Engineers, the Ghana Institute of Planners and the Ghana Real Estate Developers Association, should organise workshops and seminars to retrain and sensitise their members on the need for barrier-free designs.
- *Educational training*: The GES should encourage institutions, such as the polytechnics and universities that train practitioners of the built environment, to introduce courses on universal design in their curriculum. This will equip their students for their subsequent professional lives.

## Conclusion

The future of every community or country is determined by the level of education of its citizens, of which persons with disabilities form part. From the findings of this study, it is evident that the access needs of persons with disabilities in the 21 secondary schools were barely considered in the design and construction of the schools. This anomaly was partly resulted from the fact that most of the buildings surveyed were constructed before the passage of the PDA in 2006 (Republic of Ghana 2006). The generally low proportion of accessibility of the various elements has had negative implications for students with disabilities who attend some of these good secondary schools in Ghana. It is therefore not surprising that most persons with disability end up begging for alms on the streets and in market areas. Integration of people with disabilities into main senior secondary education system poses lot of challenges because of the huge deficiencies in the built environment that hamper smooth academic work. With the present conditions of buildings and facilities in our senior high schools, persons with disability can only attend these schools with the help of permanent assistants, a situation that is neither tenable nor sustainable for most students. A change in the situation with the help of all stakeholders in government, the construction and educational systems is therefore necessary.
